# The use of mannan antigen and anti-mannan antibodies in the diagnosis of invasive candidiasis: recommendations from the Third European Conference on Infections in Leukemia

**DOI:** 10.1186/cc9365

**Published:** 2010-12-08

**Authors:** Małgorzata Mikulska, Thierry Calandra, Maurizio Sanguinetti, Daniel Poulain, Claudio Viscoli

**Affiliations:** 1 Division of Infectious Diseases, San Martino University Hospital, L.go R. Benzi, 10, Genoa, Italy; 2 Infectious Diseases Service, Department of Medicine, Centre Hospitalier Universitaire Vaudois, and University of Lausanne, rue du Bugnon 46, CH-1011 Lausanne, Switzerland; 3 Università Cattolica del S. Cuore, Institute Of Microbiology, Largo F. Vito 1, 00168 Rome, Italy; 4 Unité Inserm U799, Faculté de Médecine, Pôle Recherche, Place Verdun, F-59045, Lille cedex, France; 5 University of Genoa, Division of Infectious Diseases, San Martino University Hospital, L.go R. Benzi, 10, Genoa, Italy

## Abstract

**Introduction:**

Timely diagnosis of invasive candidiasis (IC) remains difficult as the clinical presentation is not specific and blood cultures lack sensitivity and need a long incubation time. Thus, non-culture-based methods for diagnosing IC have been developed. Mannan antigen (Mn) and anti-mannan antibodies (A-Mn) are present in patients with IC. On behalf of the Third European Conference on Infections in Leukemia, the performance of these tests was analysed and reviewed.

**Methods:**

The literature was searched for studies using the commercially available sandwich enzyme-linked immunosorbent assays (Platelia™, Bio-Rad Laboratories, Marnes-la-Coquette, France) for detecting Mn and A-Mn in serum. The target condition of this review was IC defined according to 2008 European Organization for Research and Treatment of Cancer/Mycoses Study Group criteria. Sensitivity, specificity and diagnostic odds ratios (DOR) were calculated for Mn, A-Mn and combined Mn/A-Mn testing.

**Results:**

Overall, 14 studies that comprised 453 patients and 767 controls were reviewed. The patient populations included in the studies were mainly haematological and cancer cases in seven studies and mainly intensive care unit and surgery cases in the other seven studies. All studies but one were retrospective in design. Mn sensitivity was 58% (95% confidence interval [CI], 53-62); specificity, 93% (95% CI, 91-94) and DOR, 18 (95% CI 12-28). A-Mn sensitivity was 59% (95% CI, 54-65); specificity, 83% (95% CI, 79-97) and DOR, 12 (95% CI 7-21). Combined Mn/A-Mn sensitivity was 83% (95% CI, 79-87); specificity, 86% (95% CI, 82-90) and DOR, 58 (95% CI 27-122). Significant heterogeneity of the studies was detected. The sensitivity of both Mn and A-Mn varied for different *Candida *species, and it was the highest for *C. albicans*, followed by *C. glabrata *and *C. tropicalis*. In 73% of 45 patients with candidemia, at least one of the serological tests was positive before the culture results, with mean time advantage being 6 days for Mn and 7 days for A-Mn. In 21 patients with hepatosplenic IC, 18 (86%) had Mn or A-Mn positive test results at a median of 16 days before radiological detection of liver or spleen lesions.

**Conclusions:**

Mn and A-Mn are useful for diagnosis of IC. The performance of combined Mn/A-Mn testing is superior to either Mn or A-Mn testing.

## Introduction

Invasive candidiasis (IC) is an important infectious complication in immunocompromised patients and is associated with severe morbidity and high mortality [1]. However, the timely diagnosis of IC remains difficult as the clinical presentation is not specific and blood cultures lack sensitivity (30-50%) and need a long incubation time [2-5]. Moreover, in patients with haematological malignancies, thrombocytopenia precludes invasive diagnostic procedures during the acute phase of infection. Thus, obtaining a microbiological diagnosis in deep tissue invasive infection, such as hepatosplenic candidiasis in patients with neutropenia, is based on ultrasound, computed tomography (CT) or magnetic resonance imaging (MRI) [6,7]. In these cases, only a presumptive diagnosis is often obtained as these images are not specific for *Candida *infection. As a consequence, microbiological markers would be extremely helpful in confirming or excluding the diagnosis of an invasive fungal disease [8].

Noninvasive, non-culture-based methods for diagnosing invasive fungal disease have been studied extensively and are now being used in daily clinical practice. The importance of serological methods has been reflected in the criteria for diagnosing invasive fungal disease, which include galactomannan and β-D-glucan as microbiological criteria for diagnosing specific fungal infection [9]. The use of circulating *Candida *antigens, metabolites and antibodies for the diagnosis of IC include the detection of mannan antigen (Mn), anti-mannan antibodies (A-Mn), enolase and arabinitol and have been reported in several studies [10-13].

In 2005, the European Conference on Infections in Leukemia (ECIL) was created by several groups, including the European Group for Blood and Marrow Transplantation, the European Organization for Treatment and Research of Cancer, the European Leukemia Net and the Immunocompromised Host Society, with the main purpose of elaborating guidelines, or recommendations, for the management of infections in leukaemia and haematopoietic stem cell transplant patients. During the third ECIL meeting held in September 2009, the performance of noninvasive diagnostic tests for fungal infections, such as galactomannan, β-D-glucan, Mn and A-Mn and cryptococcal antigen, was analysed. This paper is focused on the use of Mn antigen and A-Mn antibodies in the diagnosis of invasive candidiasis.

Mn is a major component of the *C. albicans *cell wall, composing up to 7% of the cell dry weight, and is one of the main *Candida *antigens that circulate during infection [14]. Different tests have been developed to detect Mn antigen or A-Mn antibodies in serum, and they differ significantly as far as sensitivity is concerned [15]. The methods developed to detect Mn antigen in serum include latex agglutination and immunoenzymatic assays [15]. Initial observations showed that mannanemia was preferentially observed in the absence of A-Mn antibodies and that, vice versa, high levels of A-Mn antibodies were generally not associated with mannanemia [16]. The observation of this balance between Mn epitope circulation and A-Mn antibody response in patients' serum has led to the idea that the combined detection of mannanemia and A-Mn antibodies by enzyme-linked immunosorbent assays (ELISAs) may be a useful diagnostic procedure [17,18]. Therefore, ELISAs have been developed for the detection of Mn, a major *Candida *cell wall constituent, and A-Mn and are marketed as Platelia™ *Candida *Antigen (Bio-Rad Laboratories, Marnes-la-Coquette, France) and Platelia™ *Candida *Antibody [16,19]. Nowadays, ELISA is the assay most frequently used in Europe and consequently is the one with the most scientific data published. Therefore, the aim of this study was to review the literature of the past 10 years (since the Platelia™ tests have been developed and marketed) on the use of Mn and A-Mn for diagnosing IC.

## Materials and methods

The recommendations of ECIL are based on a review of the English-language literature following a predefined methodology [20]. The quality of evidence and level of recommendation were graded according to the standard scoring system of the Infectious Diseases Society of America and the U.S. Public Health Service for rating recommendations in clinical guidelines [21]. The strength of recommendation was graded as follows: (A) good evidence to support a recommendation for use, (B) moderate evidence to support a recommendation for use, and (C) poor evidence to support a recommendation. The quality of evidence was graded as follows: (I) evidence from at least one properly randomised, controlled trial; (II) evidence from at least one well-designed clinical trial, without randomisation, from cohort or case-controlled analytic studies (preferably from more than one centre), from multiple time series or from dramatic results from uncontrolled experiments; and (III) evidence based on the opinions of respected authorities, clinical experience, descriptive studies or reports of expert committees.

### Studies and patients

All of the studies that assessed the diagnostic accuracy of Mn and/or A-Mn antibody detection using immunoenzymatic methods in any patient population, with either prospective or retrospective data collection, were eligible. The tests under evaluation were the commercially available sandwich ELISAs (Platelia™) for detecting Mn and A-Mn antibodies in serum. Studies addressing detection in other fluids are discussed briefly. Studies that used tests other than ELISA were not included in this review to minimise the problem of comparing results obtained with different assays.

The target condition of this review is candidemia and any other form of IC. The following reference standards can be used to define the target condition: autopsy or the criteria of the European Organization for Treatment and Research of Cancer and Mycoses Study group (EORTC/MSG) for defining invasive fungal infections [7,9]. According to these criteria, proven candidiasis is defined as histopathologic, cytopathologic or direct microscopic examination of a specimen obtained by needle aspiration or biopsy from a normally sterile site (other than mucous membranes). The specimen must have evidence of yeast cells or recovery of a yeast by culture of a sample obtained using a sterile procedure (including a freshly placed drain) from a normally sterile site showing a clinical or radiological abnormality consistent with an infectious process.

The definition of probable invasive, that is, hepatosplenic, candidiasis, has changed during the past 6 years. In the first version of EORTC/MSG diagnostic criteria, probable *Candida *infection was diagnosed in patients with risk factors who had small, peripheral target-like abscesses (that is, bull's-eye lesions) in liver and/or spleen demonstrated by CT, MRI or ultrasound, as well as an elevated serum alkaline phosphatase level; supporting microbiological criteria were not required for probable category [7]. On the contrary, the EORTC/MSG criteria published in 2008 defined disseminated hepatosplenic candidiasis as the presence, in high-risk patients, of characteristic lesions in the liver or spleen after an episode of candidemia within the previous 2 weeks. However, this definition is problematic because blood cultures are frequently negative in these patients, despite repeated attempts to culture a large volume of blood and each lumen of intravenous catheters.

Patients with proven or probable invasive candidiasis defined according to EORTC/MSG criteria were considered as true positive subjects with IC. Subjects without IC were considered as true negatives. Patients with possible IC, that is, the presence of highly suggestive symptoms without microbiological documentation, were not included in the assessment of the performance of the test because of the uncertainty whether they represent true or false positives.

### Search methods for identification of studies

The MEDLINE electronic database was searched with the following terms: *Candida*, candidiasis, candidemia, antigen, antibody, diagnosis, mannan antigen, anti-mannan antibodies, ELISA, and Platelia™ entered both as text word and MeSH terms if present. The literature search was performed by one of the authors (MM), and the studies published between 1 January 1998 and 9 January 2010 were considered. To identify additional studies, we entered relevant studies selected from the above sources into PubMed and then used the related articles feature and checked the reference lists of all relevant manuscripts. Additionally, review articles and abstracts from the main conferences from the past 5 years (American Society of Hematology Annual Meeting, Interscience Conference on Antimicrobial Agents and Chemotherapy, Infectious Diseases Society of America Annual Meeting, European Congress of Clinical Microbiology and Infectious Diseases and Congress on Trends in Medical Mycology) were screened for any other relevant studies. The following articles were excluded from the review: animal or *in vitro *studies, articles in languages other than English, case reports and studies that included less than 10 patients (including cases and controls).

### Statistical analysis

Our reference standard was the set of EORTC/MSG criteria. To calculate tests' accuracy and to reflect the categories that are used in clinical practice, we considered the patients with proven and probable IC as having invasive *Candida *infection (true positives) and patients without candidiasis as the control group (true negatives). This resulted in two-by-two tables: positive or negative Mn antigen, A-Mn antibody or both Mn and A-Mn in each of two groups. The data in the two-by-two tables were used to calculate sensitivity and specificity for each study, while 95% confidence intervals (95% CI) were calculated using the Freeman-Tukey test. For the number of true positives, true negatives, false positives and false negatives that were reported, all of the following were calculated: the diagnostic odds ratios (DORs) with 95% CI. In case of two-by-two tables containing zeroes, 0.5 was added to all counts in the table, which is a commonly used method to calculate an approximation of DOR [22,23]. Median values of sensitivity, specificity and DOR were calculated for all of the available studies.

Individual study results, together with overall pooled results, were presented graphically by plotting the estimates of sensitivity, specificity and DOR (and their respective 95% CIs) in forest plots. The heterogeneity of the studies was investigated using a χ^2 ^test. *P *values of 0.5 or lower were considered statistically significant.

## Results

### Literature research and description of studies

Overall, 556 literature search results were retrieved and screened for relevant information. There were 22 studies that described the use of Mn and A-Mn in various patient populations. Eight studies used tests other than ELISA and were not included in this review. Thus, 14 studies on Mn/A-Mn immunoenzymatic tests were reviewed. The description of these 14 studies is outlined in Table 1 in chronological order.

**Table 1:** Description of the studies that used Platelia™ mannan (Mn) and anti-mannan (A-Mn) assay (in reverse chronological order)

	First author, year of publication, country, type of study	Cutoff value of Mn and A-Mn, number of samples to declare positive	Underlying condition/risk factor for IC	Diagnostic criteria for *Candida *infection (number of patients with different sites of IC)	No. of patients and no. of samples	No. (%) of patients with *C. albicans*	No. of control patients and samples	Type of control group
1	Verduyn Lunel *et al*., 2009, Netherlands, retrospective [31]	Mn ≥ 0.25 ng/ml A-Mn ≥ 5 AU/ml Single sample	Chemotherapy	Culture from a sterile site	21 and 242 divided into: neutropenic for less or more than 15 days: 10 and 11, respectively	12 (57%)	30 and 390	Patients with haematological malignancies
2	Ellis *et al*., 2009, UAE, prospective [26]	Mn ≥ 0.25 ng/ml; A-Mn ≥ 2.5 AU/ml Two consecutive samples positive for both Mn and A-Mn	Haematological malignancies	IC EORTC (5 candidemia and 7 hepatosplenic IC)	12 and 216	1	74	High-risk patients without IC (50 febrile neutropenia, 24 mould infection)
3	Sendid *et al*., 2008, France, retrospective [37]	Mn ≥ 0.5 ng/ml A-Mn ≥ 10 AU/ml Single sample	Mostly ICU and surgery, 14; haematological malignancy 2.	Candidemia	18 and 69	18 (100%)	None	-
4	Oliveri *et al*., 2008, Italy, ND [24]	Mn ≥ 0.5 ng/ml Two samples	Neonatal ICU	Candidemia and probable IC defined as presence of sign and symptoms despite broad spectrum antibiotics + *Candida *colonisation	18 (12 candidemia and 6 probable IC) and 18	ND	52 and 52	Neonates from the same ward without IC
5	Alam *et al*., 2007, Kuwait, retrospective [28]	Mn ≥ 0.5 ng/ml A-Mn ≥ 10 AU/ml Single sample	Mostly ICU; 2 haematological malignancies	Candidemia	27 and 32	18 (67%)	26 and 26	10 patients with vaginal candidiasis, 16 healthy controls (39 patients with clinically suspected IC were not considered as a control group)
6	Fujita *et al*., 2006, Japan, retrospective [29]	Mn ≥ 0.5 ng/ml Single sample	Solid tumour, 69; haematological malignancy, 8; other, 28	Candidemia	105 and 251	49 (33%)	175 and 178	Febrile patients with or without bacteraemia
7	Prella *et al*., 2005, Switzerland, retrospective [25]	Mn ≥ 0.25 ng/ml A-Mn ≥ 5 AU/ml Two samples	Haematological malignancies	IC proven and probable according to EORTC (12 candidemia, 14 hepatosplenic IC)	26 and ND	5 (19%)	25 and 163	Patients with haematological malignancy and noncandidal infection
8	White *et al*., 2005, UK, retrospective [32]	Mn ≥ 0.5 ng/ml Single sample	Haematological malignancies, 14; other, 6	IC EORTC for haematological patients and culture or underlying condition + signs and symptoms + colonisation for nonhaematological (2 proven, 13 probable hepatosplenic and 5 probable in non haematological)	20 and ND	ND	67 and ND	High-risk patients (not included 18 haematology patients with possible IC)
9	Sendid *et al*., 2004, France, retrospective [33]	Mn ≥ 0.5 ng/ml Single sample	Mostly ICU and surgery, 21; haematological malignancy, 3; other, 2.	Signs and symptoms + culture (19 candidemia, other culture sites included BAL in 5, bronchial biopsy and pleural liquid in 1)	26 and 90	18 (69%)	118 and 148	70 healthy donors, 10 patients with IFD, 24 high risk patients, mostly ICU, 14 subjects with high rheumatoid factor titres
10	Sendid *et al*., 2003, France, retrospective [27]	Mn ≥ 0.5 ng/ml A-Mn ≥ 10 AU/ml Single sample	Haematological malignancies	Candidemia due to *C. tropicalis*	7 and 82	0	12 and 48	Febrile neutropenic patients without candidemia
11	Sendid *et al*., 2002, retrospective [17]	Mn ≥ 0.5 ng/ml A-Mn ≥ 10 AU/ml Single sample	Mostly ICU and surgery, 41; haematological malignancies, 10; other, 12	Signs and symptoms + culture (58 candidemia, 2 peritoneum cultures, 2 spleen cultures)	63 and 204	21 (33%) and *C. glabrata*, 12; *C. tropicalis*, 10; *C. parapsilosis*, 10; *C. krusei*, 8	None	-
12	Persat *et al*., 2002, France, retrospective [34]	Mn ≥ 0.5 ng/ml A-Mn ≥ 10 AU/ml Single sample	Cancer, 7; haematological malignancy, 6; surgery, 2; other, 7	IC EORTC	22 and 22	14 (64%)	38 and 38	10 healthy individuals, 10 patients at risk but without IC, 18 with Candida colonisation
13	Yera *et al*., 2001, France, retrospective [18]	Mn ≥ 0.5 ng/ml A-Mn ≥ 10 AU/ml Single sample	ICU and surgery, 32; haematological malignancies, 11; other, 2	Candidemia	45 and 137	23 (51%)	None	-
14	Sendid *et al*., 1999, France, retrospective [16]	Mn ≥ 0.5 ng/ml A-Mn ≥ 10 AU/ml Single sample	ICU and surgery, 32; haematological malignancy, 1, other, 10	Signs and symptoms + culture from a sterile site (23 candidemia, 14 surgery drain cultures)	43 and 162	43 (100%)	150 and 230	98 healthy blood donors and 52 hospitalised patients without IC (of them 29 with IFD: 12 IA, 13 cryptococcosis and 4 PCP)

UAE, United Arab Emirates; Ab, antibody; Ag, antigen; IC, invasive candidiasis; IA, invasive aspergillosis; IC, invasive candidiasis; ICU, intensive care unit; IFD, invasive fungal disease; ND, no data; PCP, *Pneumocystis jiroveci *pneumonia.

The number of patients included in the studies varied from 7 to 105, with a median of 25 patients per study. Four studies were performed exclusively in patients with haematological malignancies, three studies were conducted in patients mostly with cancer or haematological malignancy and the remaining seven studies included mostly or exclusively patients from intensive care unit (ICU) or surgery wards (among them was one study from a neonatal ICU). Overall, among 453 case patients described, 123 (27%) had haematological disorders. A control group was included in only 11 of 14 studies and most frequently consisted of patients with similar risk factors, but without IC and sometimes with other documented infections. Four studies included healthy blood donors as control samples.

All of the studies performed included Mn antigen testing, while only 11 of them also searched for A-Mn antibodies. Thus, the sensitivity of the test could be evaluated in all studies (14 for Mn, 10 for A-Mn and combined Mn/A-Mn), but the specificity could be evaluated in only 11 studies that included a control group. In all 11 studies, Mn specificity was evaluated, while the specificity of A-Mn or combined Mn/A-Mn testing was reported in only 7 and 6 papers, respectively.

For Platelia™ Mn antigen and A-Mn antibody testing, the values of 0.5 ng/mL for Mn and 10 arbitrary units (AU)/mL for A-Mn are defined as positive according to the manufacturer, while the values 0.25-0.5 ng/mL for Mn and 5-10 AU/mL for A-Mn were considered indeterminate. Most studies defined a positive result according to the cutoff value recommended by the manufacturer in a single serum sample. In two studies, a result was regarded as positive if in two samples Mn or A-Mn or Mn in one and A-Mn in one were above intermediate cutoff thresholds [24,25]. Additionally, one prospective study differed significantly as far as sampling and threshold values are concerned. In the study by Ellis *et al*. [26], the cutoff used for A-Mn was two to four times lower than the others (2.6 vs. 5 or 10), but different criteria were used to define a positive Mn/A-Mn result, that is, two consecutive samples positive for both Mn and A-Mn. Therefore, for A-Mn and Mn/A-Mn testing, the results obtained in this way are reported. For each study, the cutoff values used are reported in Table 1.

### Sensitivity, specificity and diagnostic odds ratio

**Figure 1:**
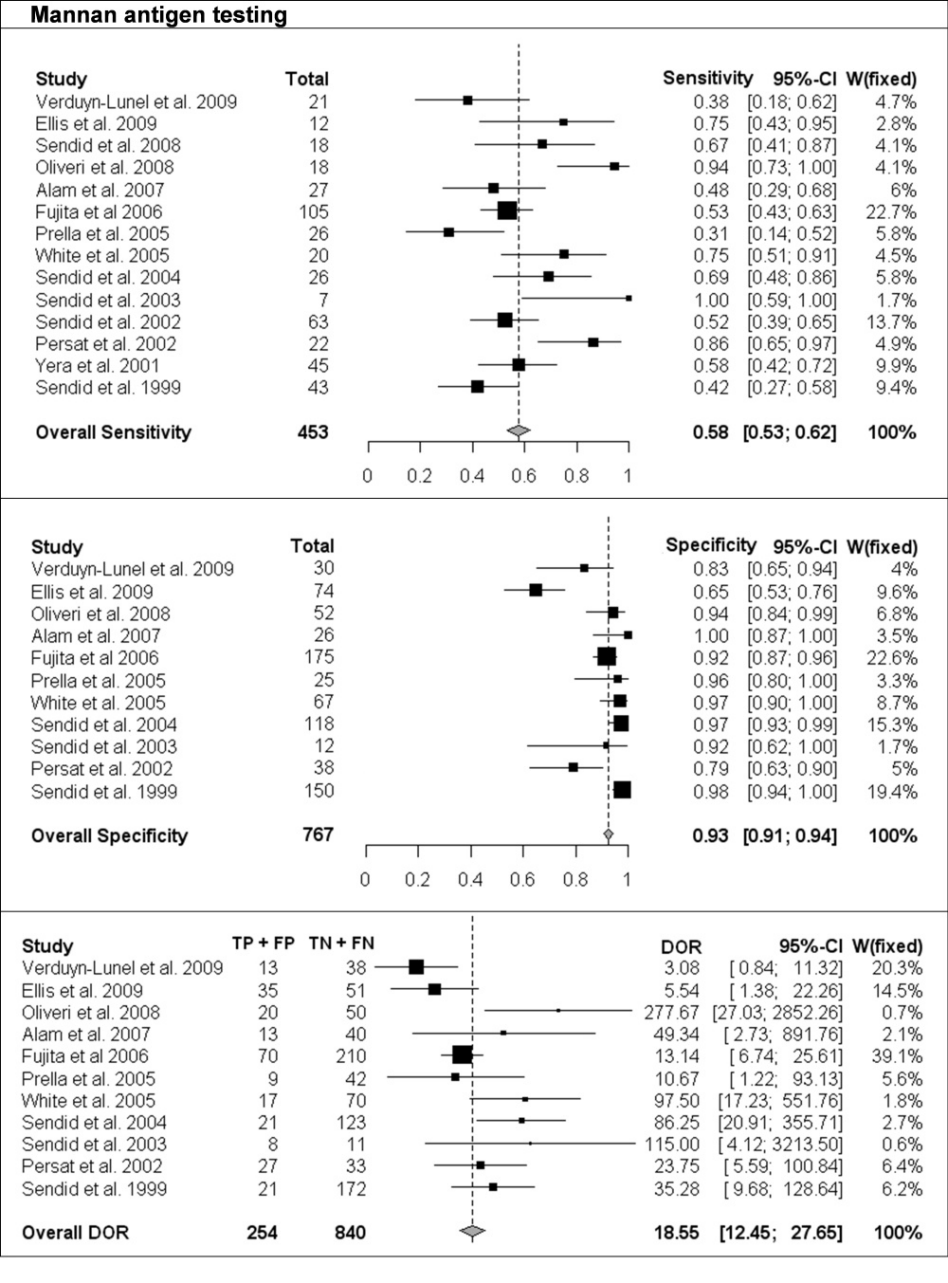
**Single-study and overall sensitivity, specificity and diagnostic odds ratio (DOR) for mannan antigen testing**. Total number of patients and a weight of each single study in meta-analysis are reported.

**Figure 2:**
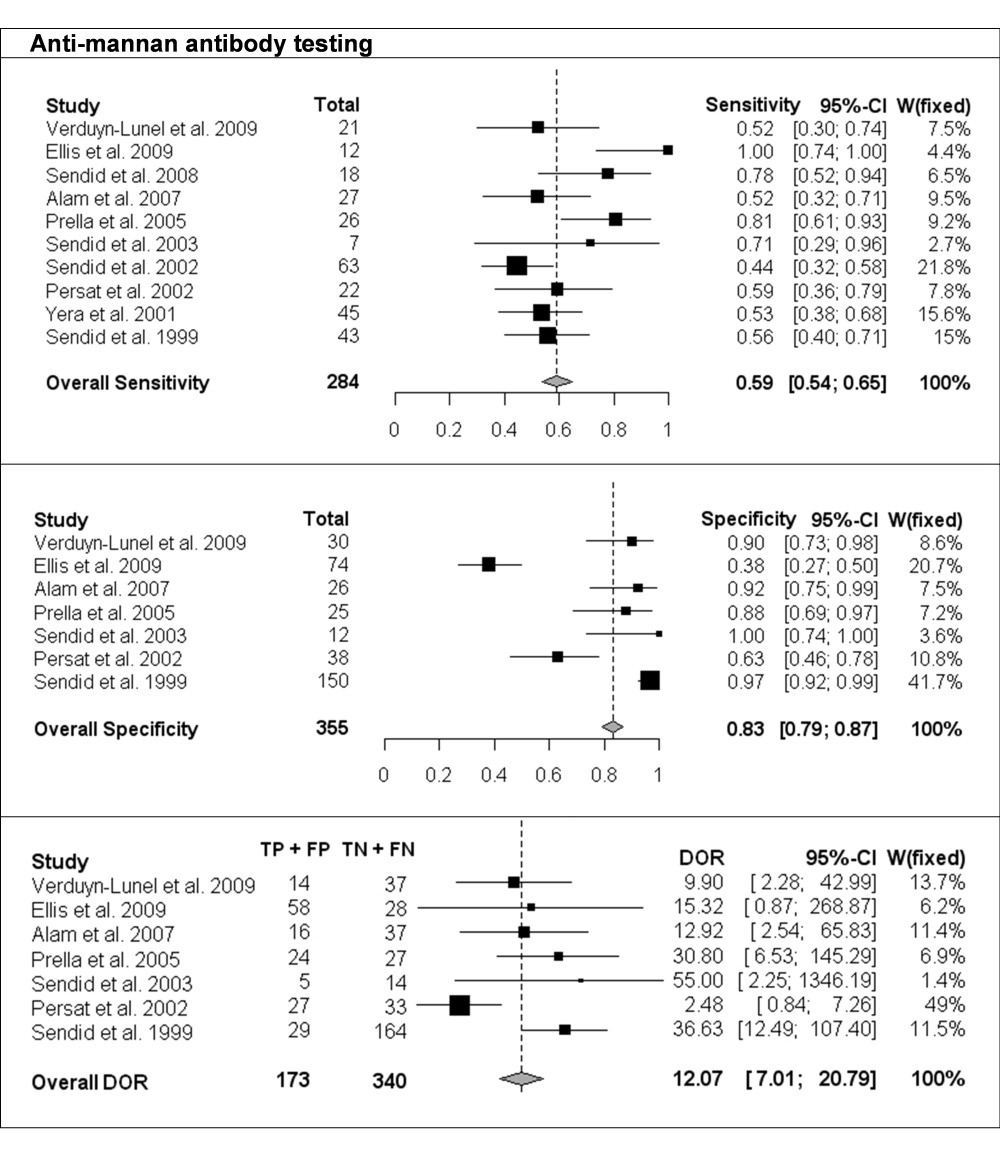
**Single-study and overall sensitivity, specificity and DOR for anti-mannan antibody testing**. Total number of patients and a weight of each single study in meta-analysis are reported.

**Figure 3:**
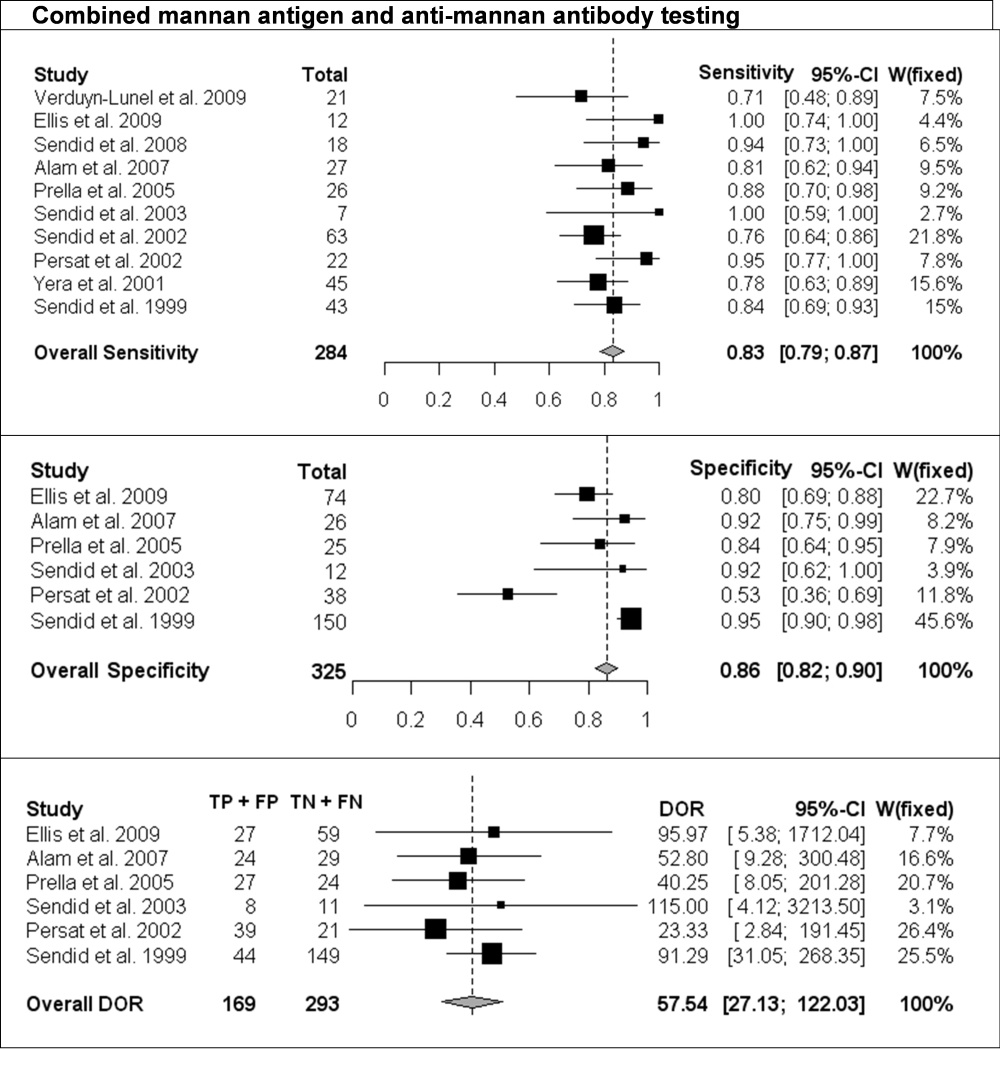
**Single-study and overall sensitivity, specificity and DOR for combined mannan antigen and anti-mannan antibody testing**. Total number of patients and a weight of each single study in meta-analysis are reported.

The per-patient values of sensitivity and specificity (with 95% CI) with respect to the reference diagnostic method of Mn, A-Mn and combined Mn/A-Mn testing are reported in Table 2. The overall pooled results, together with the results of single studies and their respective weight in meta-analysis, are reported as forest plots in Figures 1, 2 and 3 for Mn, A-Mn and combined Mn/A-Mn testing, respectively.

**Table 2:** Per-patient sensitivity, specificity and diagnostic odds ratio (DOR), with 95% confidence intervals of mannan antigen (Mn), anti-mannan antibodies (A-Mn) and combined Mn/A-Mn testing for separate studies, median of the studies and totala

Study	Sensitivity (95% CI), absolute numbers: true positives/total			Specificity (95% CI), absolute numbers: true negatives/total			DOR (95% CI)		
			
	Mn	A-Mn	Mn/A-Mn	Mn	A-Mn	Mn/A-Mn	Mn	A-Mn	Mn/A-Mn
1. Verduyn Lunel *et al*., 2009 [31]	0.38 (0.18-0.62), 8/21	0.52 (0.30-0.74), 11/21	0.71 (0.48-0.89), 15/21	0.83 (0.65-0.94), 25/30	0.90 (0.73-0.98), 27/30	-	3.1 (0.8-11.3)	9.9 (2.3-43)	-
2. Ellis *et al*., 2009 [26]^b ^	0.75 (0.43-0.95), 9/12	1.00 (0.74-1.00), 12/12	1.00 (0.74-100), 12/12	0.65 (0.53-0.76), 48/74	0.38 (0.27-0.50), 28/74	0.80 (0.69-0.88), 59/74	5.5 (1.4-22.3)	15.3 (0.9-268.9)	96 (5.4-1712)
3. Sendid *et al*., 2008 [37]	0.67 (0.41-0.87), 12/18	0.78 (0.52-0.94), 14/18	0.94 (0.73-0.99), 17/18	-	-	-	-	-	-
4. Oliveri *et al*., 2008 [24]	0.94 (0.73-0.99), 17/18	-	-	0.94 (0.84-0.99), 49/52	-	-	277.7 (27-2852.3)	-	-
5. Alam *et al*., 2007 [28]	0.48 (0.29-0.68), 13/27	0.52 (0.32-0.71), 14/27	0.81 (0.62-0.94), 22/27	1.00 (0.87-1.00), 26/26	0.92 (0.75-0.99), 24/26	0. 92 (0.75-0.99), 24/26	49.3 (2.7-891.8)	12.9 (2.5-65.8)	52.8 (9.3-300.5)
6. Fujita *et al*., 2006 [29]	0.53 (0.43-0.63), 56/105	-	-	0.92 (0.87-0.96), 161/175			13.1 (6.7-25.6)	-	-
7. Prella *et al*., 2005 [25]	0.31 (0.14-0.52), 8/26	0.81 (0.61-0.93), 21/26	0.88 (0.70-0.98), 23/26	0.96 (0.80-0.99), 24/25	0.88 (0.69-0.97), 22/25	0.84 (0.64-0.95), 21/25 (all 4 colonised)	10.7 (1.2-93.1)	30.8 (6.5-145.3)	40.3 (8.1-201.3)
8. White *et al*., 2005 [32]	0.75 (0.51-0.91), 15/20	-	-	0.97 (0.90-0.99), 65/67	-	-	97.5 (17.2-551.8)	-	-
9. Sendid *et al*., 2004 [33]	0.69 (0.48-0.86), 18/26	-	-	0.97 (0.93-0.99), 115/118	-	-	86.3 (20.9-355.7)	-	-
10. Sendid *et al*., 2003 [27]	1.00 (0.59-1.00), 7/7	0.71 (0.29-0.96), 5/7	1.00 (0.59-1.00), 7/7	0.92 (0.62-0.99), 11/12	1.00 (0.74-1.00), 12/12	0.92 (0.62-0.99), 11/12	115 (4.1-3213.5)	55 (2.2-1346,2)	115 (4.1-3213.5)
11. Sendid *et al*., 2002 [17]	0.52 (0.39-0.65), 33/63	0.44 (0.32-0.58), 28/63	0.76 (0.64-0.86), 48/63	-	-	-	-	-	-
12. Persat *et al*., 2002 [34]	0.86 (0.65-0.97), 19/22	0.59 (0.36-0.79), 13/22	0.95 (0.77-0.99), 21/22	0.79 (0.63-0.90), 30/38	0.63 (0.46-0.78), 24/38	0.53 (0.36-0.69), 20/38	23.8 (5.6-100.8)	2.48 (0.9-7.3)	23.3 (2.8-191.5)
13. Yera *et al*., 2001 [18]	0.58 (0.42-0.72), 26/45	0.53 (0.38-0.68), 24/45	0.78 (0.63-0.89), 35/45	-	-	-	-	-	-
14. Sendid *et al*., 1999 [16]	0.42 (0.27-0.58), 18/43	0.56 (0.40-0.71), 24/43	0.84 (0.69-0.93), 36/43	0.98 (0.94-0.99), 147/150	0.97 (0.92-0.99), 145/150	0.95 (0.90-0.98), 142/150	35.3 (9.7-128.6)	36.6 (12.5-107.4)	91.3 (31.1-268.4)
Median of all the studies (range)	0.62 (0.31-1.0)	0.57 (0.44-1.0)	0.86 (0.71-1.0)	0.94 (0.65-1.0)	0.9 (0.38-1.0)	0. 88 (0.53-0.92)			
Pooled overall	0.58 (0.53-0.62), 259/453	0.59 (0.54-0.65), 166/284 ^2^	0.83 (0.79-0.87), 236/284^c^	0.93 (0.91-0.94), 701/767^d^	0.83 (0.79-0.87), 282/355^e^	0.86 (0.82-0.90), 277/325^f^	18.6 (12.5-27.7)^d^	12.1 (7-20.8)^e^	57.5 (27.1-122)^f^

^a^NR, not reported; -, no data. ^b^Absolute numbers calculated on basis of reported percentage sensitivity and specificity values. ^c^Available for 10 studies. ^d^Data from 11 studies. ^e^Data from 7 studies. ^f^Data from 6 studies.

The median sensitivity of all the studies was 62%, ranging from 31% in the study by Prella *et al*. [25] to 100% in the study by Sendid *et al*. that reported seven cases of candidemia due to *C. tropicalis *[27]. The overall pooled per-patient sensitivity of Mn was evaluated in 14 studies in 453 patients and resulted in 58% sensitivity (95% CI, 53-62). Antibody testing was performed in 10 studies with a total of 284 patients, and the sensitivity of the antibody assay was 59% (95% CI, 54-65), with the median value of 57%, ranging from 44% to 100% [17,26]. The median sensitivity of combined Mn/A-Mn testing (that is, when either Mn- or A-Mn-positive results were considered, except for the study by Ellis *et al*. [26], in which a different definition of positivity was used as reported above) was 86%, ranging from 71% to 100%. The pooled overall sensitivity of Mn/A-Mn was 83% (95% CI, 79-87).

Eleven studies included a control group, allowing the assessment of specificity and the calculation of DOR. The specificity of Mn testing was performed in all 11 studies and resulted in a range from 65% in the study by Ellis *et al*. [26] to 100% in the study reported by Alam *et al*. [28], with an overall pooled specificity of 93% (95% CI, 91-94). For A-Mn testing, the specificity was evaluated in seven studies and with a pooled overall result of 83% (95% CI, 79-97), ranging from 38% to 100% (the lowest value was reported in the study by Ellis *et al*. [26]). The overall specificity of combined Mn/A-Mn assay was 86% (95% CI, 82-90).

The DORs were calculated for Mn, A-Mn and Mn/A-Mn testing and differed significantly between the studies (Figures 1, 2, 3). Overall, DOR was the highest in the case of combined Mn/A-Mn testing (58; 95% CI, 27-122), followed by Mn testing (18; 95% CI, 12-28) and A-Mn (12; 95% CI, 7-21).

Significant heterogeneity of the studies was detected for the sensitivity of Mn (*P *< 0.0001) and A-Mn (*P *= 0.0002); the specificity of Mn, A-Mn and Mn/A-Mn (*P *< 0.0001 for all); and the DORs of Mn (*P *= 0.004) and A-Mn (*P *= 0.01). To reduce the heterogeneity, the same pooled values were calculated with the exclusion of the one study that differed significantly from the others, that is, the study by Ellis *et al*. [26]. However, even with this study excluded, the heterogeneity remained significant (data not shown). When the studies were grouped by underlying disease (haematological or cancer and ICU or surgery), the heterogeneity disappeared in some of the subgroups, but this effect might be due to the low number of studies included in each subgroup (data not shown). Therefore, the final overall pooled results are reported for all the studies available (Figures 1, 2, 3).

Additionally, per-sample values were reported in five studies in which there were more samples than patients. The per-sample values were not considered significantly different from per-patient data for the study by Alam *et al*. [28], where only five patients had two samples instead of one. The overall per-sample sensitivity was lower than the per-patient sensitivity, but the specificity remained high (Table 3).

**Table 3:** Per sample sensitivity, specificity, with 95% confidence intervals of Mn, A-Mn and combined Mn/A-Mn testing

Study	Mn	A-Mn	Mn/A-Mn	Mn	A-Mn	Mn/A-Mn
Verduyn Lunel, 2009 [31]	0.17 (0.13-0.22), 41/240	0.39 (0.33-0.45), 93/238	-	0.95 (0.92-0.97), 379/390	0.87 (0.83-0.90), 347/384	-
Sendid, 2008 [37]	0.67 (0.55-0.77), 46/69	0.35 (0.25-0.47), 24/69	-	-	-	-
Fujita, 2006 [29]	0.45 (0.39-0.51), 112/251	-	-	0.92 (0.87-0.95), 164/178		
Sendid *et al*., 2003 [27]	0.54 (0.43-0.64), 44/82	0.23 (0.15-0.33), 19/82	0.68 (0.58-0.77), 56/82	0.98 (0.89-0.99), 47/48	1 (0.93-1.0), 48/48	0.98 (0.89-0.99), 47/48
Sendid *et al*., 2002 [17]	0.35 (0.29-0.42), 72/204	0.27 (0.22-0.34), 56/204	0.55 (0.49-0.62), 113/204	-	-	-
Sendid *et al*., 1999 [16]	0.27 (0.20-0.34), 43/162	0.39 (0.32-0.47), 63/162	0.62 (0.55-0.69), 101/162	0.99 (0.96-0.99), 227/230	0.96 (0.93-0.98), 221/230	0.94 (0.91-0.97), 218/230
Median of all the studies (range)	0.40 (0.17-0.67)	0.35 (0.23-0.39)	0.62 (0.55-0.68)	0.97 (0.92-0.99)	0.96 (0.87-1.0)	0.96 (0.94-0.98)

-, no data.

### Different *Candida *species

The sensitivity of both Mn and A-Mn varied for different *Candida *species, and it was the highest for *C. albicans*, followed by *C. glabrata *and *C. tropicalis *[27,29]. In particular, according to the results reported by Sendid *et al*. [17], the sensitivity for the detection of Mn was 58%-70% for infections caused by *C. albicans*, *C. glabrata *and *C. tropicalis*, while it was 25%-30% for infections caused by *C. parapsilosis *and *C. krusei *(Table 4). The difference was even more pronounced in the study by Fujita *et al*. [29], where the sensitivity for *C. albicans *was 78% compared to 15% and 0 for *C. parapsilosis *and *C. krusei*, respectively. Even though the sensitivity varied among the studies, it was clearly lower in cases of *C. parapsilosis *and *C. krusei*, probably because of the lower amount of Mn produced and released by these species [19,30].

**Table 4:** Sensitivity of Mn and/or A-Mn testing in different Candida species

Species	Study	Number of isolates	Sensitivity		
			
			Mn	A-Mn	Mn/A-Mn
*C. albicans*	Fujita *et al*., 2006 [29]	49	78%		
	Sendid et al., 2002 [17]	21	62%	67%	100%
*C. tropicalis*	Sendid *et al*., 2002 [17]	10	70%	60%	80%
	Fujita *et al*., 2006 [29]	9	67%		
	Sendid *et al*., 2003 [27]	7	100%	71%	100%
*C. glabrata*	Sendid *et al*., 2002 [17]	12	58%	83%	83%
	Fujita *et al*., 2006 [29]	11	36%		
*C. guilliermondi*	Fujita *et al*., 2006 [29]	11	27%		
*C. parapsilosis*	Fujita *et al*., 2006 [29]	20	15%		
	Sendid *et al*., 2002 [17]	10	30%	10%	40%
*C. krusei*	Sendid *et al*., 2002 [17]	8	25%	38%	50%
	Fujita *et al*., 2006 [29]	2	0		

### Timing of diagnosis

Another point worth analysing while reviewing studies on non-culture-based diagnostic methods is the time to diagnosis of IC compared to traditional methods. This advantage in early diagnosis was reported in five studies in both haematological and ICU patients [17,18,24,25,31]. In 73% of 45 patients with candidemia, at least one of the serological tests was positive before the culture results [18], and in patients in whom the Mn and/or A-Mn antibody tests were positive before blood culture, the mean time advantage was 6 days for Mn and 7 days for A-Mn. These findings were confirmed in another study of 63 patients, in whom at least one of the serological tests was positive before yeast growth occurred in 60% of patients for whom a serum sample was available before blood culture sampling and an increase in serological test positivity to 85% was observed for sera obtained on the date of positive culture, irrespective of the *Candida *species isolated [17]. Similarly, in a recent study of patients undergoing chemotherapy, serological tests were positive significantly earlier than culture, that is, in a median of 23 days for A-Mn and 1 day earlier for Mn [31]. Even in the neonatal ICU, Mn could be detected before the day of blood sampling in 8 of 12 patients with proven IC, with the time advantage of 8.5 days [24]. Last but not least, in 21 patients with hepatosplenic lesions highly suggestive of candidiasis, 18 (86%) had positive Mn and/or A-Mn antibody tests at a median of 16 days before radiological detection of liver or spleen lesions [25]. In fact, the study by Prella *et al*. [25] was the first one to show the usefulness of Mn and A-Mn serum level determination in patients with suspected hepatosplenic IC, allowing the diagnosis of this complication before neutrophil recovery in the majority of patients. The clinical utility of serological testing in this setting was confirmed by the study of Ellis *et al*., in which 7 of 12 patients with IC had the hepatosplenic form [26].

### Possible invasive candidiasis and colonisation

Obviously, the most interesting patients are those with possible IC, where culture is probably not sensitive enough to detect candidemia and where a more sensitive method, such as antigen testing, might prove extremely helpful. The fact that Mn is more sensitive than culture is indirectly proved by the fact that Mn sensitivity in groups of patients with possible candidemia is higher than that in controls but lower than that in culture-positive IC. For example, in the study by White *et al*. [32], 5 of 18 patients with possible *Candida *infection had positive results on Mn testing. Similarly, in 39 patients with clinically suspected IC, Mn and A-Mn were present in 16% and 29% of patients, respectively [28].

The colonisation with *Candida*, particularly if multiple sites are colonised, has always been feared to be a potential reason for the lower specificity of Mn or A-Mn testing. Indeed, lower specificities are generally observed in colonised subjects, and *Candida *colonisation has been reported to result in detectable A-Mn antibody levels in approximately 30% of uninfected patients [16]. Therefore, we reviewed the data on test performance in patients with *Candida *colonisation.

Overall, four studies included patients with *Candida *colonisation in their control population. In the study by Verduyn Lunel *et al*. [31], 19 of 21 patients and 20 of 30 controls were colonised with *Candida *(mostly *C. albicans*) as detected by two consecutive samples from mouthwashes and/or faeces. However, in the logistic regression analysis, neither prior colonization nor superficial *Candida *infections were associated with the detection of Mn or A-Mn. On the contrary, A-Mn was detected in patients with *Candida *colonisation in the study by Sendid *et al*. [16], in which one of the control groups comprised 23 ICU patients, of whom 19 had *Candida *colonisation. In this group, only one patient (4%) had positive Mn, but 6 (26%) of 19 had positive A-Mn results [16]. Similarly, in a control group of 10 patients with vaginal candidiasis, 2 patients (20%) had a positive Mn result [28], while among 15 ICU patients colonised with *Candida *at two sites or more, only 1 patient (7%) had a positive A-Mn result [33]. Higher rates of false positives were reported in the study by Persat *et al*. [34], where 18 of 38 control patients had *Candida *colonisation, 4 (22%) had positive results for Mn and 8 (44%) had positive results for A-Mn. Finally, in the study by Ellis *et al*. [26], where 60% of 74 control patients had *Candida *colonisation, the specificities of both Mn and A-Mn were significantly lower than reported in other studies. In particular, the specificity was only 21% if two consecutive positive results for either Mn or A-Mn were evaluated [26]. Such a low specificity differs from the results of the other studies and may be related to the fact that a particularly low cutoff value was used for A-Mn testing.

Even though ELISA is licensed to be used in serum only, Verduyn Lunel *et al*. [35] reported an interesting use of Mn testing in cerebrospinal fluid (CSF) in five patients with *Candida *meningitis. In fact, four of them tested positive for Mn in CSF. Additionally, a recent study performed in preterm infants found that Mn detection in bronchoalveolar lavage fluid might be useful for early identification and preemptive treatment of candidemia in these patients [36].

## Discussion

The review of the use of Mn and A-Mn in patients with confirmed or suspected IC showed that these noninvasive tests might be useful for microbiological confirmation or exclusion of the diagnosis of IC. The overall performance of combined Mn/A-Mn testing was superior to either Mn or A-Mn testing alone.

In most of the studies, the diagnostic performance of Mn and A-Mn tests was compared to blood culture as a gold standard, and they were positive before the results of the latter, thus allowing for earlier diagnosis of IC. Despite the fact that prompt diagnosis and treatment are crucial for prognosis in IC, these tests are not intended to replace blood cultures, and special consideration for their use concerns the 40%-50% of patients with IC in whom blood cultures remain constantly negative. There is no reason why the specificity for IC, established by comparison with blood culture, could not apply to the patients with negative blood cultures. Thus, for patients with significant mannanemia or A-Mn antibodies, antifungal treatment might be considered.

Even though high overall specificity and sensitivity were found in the aforementioned studies, the optimal way to use these tests in daily clinical practice remains to be defined. In fact, only one study was prospective, and the results obtained differed importantly from other studies [26]. Whereas numerous factors might have been responsible for the low specificity reported by Ellis *et al*. [26], only further prospective studies will define the strategies of Mn/A-Mn testing for diagnosis of candidemia in times when β-D-glucan use is becoming more and more popular. In particular, Mn/A-Mn testing might be seen as complementary in cases with a positive β-D-glucan result, given that β-D-glucan is nonspecific. In such cases, positive Mn or A-Mn results might indicate fungal disease due to *Candida*, while a negative Mn/A-Mn test could indicate infection caused by other fungi. The utility of such an approach should be investigated.

Another aspect of Mn/A-Mn testing is its utility in diagnosing hepatosplenic candidiasis in neutropenic patients who do not yet show evidence of radiological lesions because of the absence of neutrophils. Mn/A-Mn testing might provide a valid clue to the aetiology of fever in such infections. Considering that the sensitivity is highest for *C. albicans *and *C. tropicalis *species, this approach seems the most promising in patients who do not receive fluconazole prophylaxis and thus are at risk for infections caused by species other than *C. krusei *or *C. glabrata*.

Several limitations of this review have to be acknowledged. First, despite the fact that we included studies conducted more than 10 years ago, the number of studies is limited, and publication bias, that is, reporting only the results of good performance of a diagnostic test, might be present. Second, only one of the studies is prospective in design [26]; thus more studies are warranted to evaluate the clinical everyday utility of a single positive result. Third, the studies analysed were quite heterogeneous as far as patient population was concerned. Indeed, some studies included patients from the ICU and surgery, while others concentrated on those with haematological malignancies. It is true that these are two entirely different groups that require different management strategies, including, for example, the administration of antifungal prophylaxis and the possibility of postponing therapy. Moreover, control groups were not included in some studies, while in others they differed from healthy individuals to patients at high risk for candidemia but with negative blood cultures. However, in 7 of 11 studies, the control population included patients with exactly the same underlying condition as the study cases, and none of the studies considered only healthy individuals as controls. Fourth, different cutoff values were used, even though the thresholds of 2.5 mg/ml Mn and 5 AU/ml for A-Mn were used most frequently. Last but not least, the sampling and criteria used for defining a positive case varied between the studies, with some regarding a result as positive only if two tests were above the cutoff value. On the other hand, the advantages of these assays include no need for invasive procedures; good sensitivity and specificity; standardised, simple and commercially available kits; and affordable costs. Therefore, even though the design of the studies was not uniform, the reported results are encouraging, and considering the increasing interest and importance of noninvasive, non-culture-based procedures in diagnosing fungal disease, Mn/A-Mn testing might offer substantial help to clinicians caring for high-risk patients.

Prospective studies are warranted to confirm the advantages of Mn and A-Mn testing in everyday clinical practice. Different populations who are at high risk of developing IC, such as patients with haematological malignancies, patients admitted to the ICU or those who have undergone abdominal surgery, should be studied separately to draw reliable conclusions about the positive and negative predictive value of a single or multiple positive results. Moreover, randomised, prospective studies might confirm benefits in terms of outcome if preemptive antifungal treatment is started early on the basis of positive Mn or A-Mn results.

## Conclusions

On the basis of the literature review, Mn antigen and A-Mn antibody offer diagnostic help in patients with suspected IC. Therefore, the following recommendations have been made by the Third European Conference on Infections in Leukemia (ECIL-3) members: the use of combined Mn/A-Mn is preferred over Mn or A-Mn alone for diagnosing invasive *Candida *infection, BII; combined Mn/A-Mn testing is useful for supporting the diagnosis of candidemia, CII; and combined Mn/A-Mn testing is useful for diagnosing hepatosplenic candidiasis, BIII.

## Key messages

• Diagnosis of IC is difficult in high-risk patients, thus noninvasive tests that detect *Candida *components in the serum of patients with IC have been developed.

• Performance of Mn and A-Mn antibody tests was analysed and reviewed on behalf of ECIL-3.

• Overall, 14 studies that included haematological malignancy and ICU patients were reviewed.

• Moderate sensitivity and good specificity of Mn and A-Mn were found (Mn, 58% and 93%; A-Mn, 59% and 83%, respectively).

• Combined Mn/A-Mn testing was better than each test alone (sensitivity 83% and specificity 86%).

• Combined Ma/A-Mn testing improves the diagnosis of IC in ICU or surgery and haematology patients.

## Abbreviations

A-Mn: anti-mannan antibodies; AU: arbitrary units; CI: confidence interval; CSF: cerebrospinal fluid; CT: computed tomography; DOR: diagnostic odds ratio; ECIL: European Conference on Infections in Leukemia; ELISA: enzyme-linked immunosorbent assay; EORTC/MSG: European Organization for Treatment and Research of Cancer and Mycoses Study group; IC: invasive candidiasis; ICU: intensive care unit; Mn: mannan antigen; MRI: magnetic resonance imaging.

## Competing interests

TC has received research grants and honoraria and has served as a consultant for Merck, Pfizer, Novartis, bioMérieux, Bio-Rad and Astellas Pharma. DP has received research grants and is a consultant for Bio-Rad. All other authors declare that they have no competing interests.

## Authors' contributions

MM performed the literature search, participated in the design of the study and analysis of the results, and drafted the manuscript. TC participated in the design of the study and helped to draft the manuscript. MS participated in the design of the study and helped to draft the manuscript. DP participated in the design of the study and helped to draft the manuscript. CV conceived of the study, participated in the design of the study and helped to draft the manuscript. All authors read and approved the final manuscript.

## Acknowledgements

The authors are indebted to Maria Pia Sormani and Alessio Signori for providing help with statistical analysis. They are also indebted to the participants of the ECIL-3 meeting: Murat Akova, Turkey; Maiken Arendrup, Denmark; Rosemary Barnes, UK; Jacques Bille, Switzerland; Stephane Bretagne, France; Thierry Calandra, Switzerland; Elio Castagnola, Italy; Catherine Cordonnier, France; Oliver A Cornely, Germany; Mario Cruciani, Italy; Manuel Cuenca-Estrella, Spain; Eric Dannaoui, France; Rafael De La Camara, Spain; Emma Dellow (Gilead Sciences), UK; Peter Donnelly, The Netherlands; Lubos Drgona, Slovakia; Hermann Einsele, Germany; Dan Engelhard, Israel; Ursula Flückiger, Switzerland; Bertrand Gachot, France; Jesus Gonzales-Moreno (Merck Sharp Dohme), Spain; Andreas Groll, Germany; Ina Hanel (Astellas), Germany; Raoul Herbrecht, France; Claus-Peter Heussel, Germany; Brian Jones, UK; Christopher Kibbler, UK; Nikolai Klimko, Russia; Lena Klingspor, Sweden; Michal Kouba, Czech Republic; Frederic Lamoth, Switzerland; Fanny Lanternier, France; Thomas Lehrnbecher, Germany; Juergen Loeffler, Germany; Olivier Lortholary, France; Johan Maertens, Belgium; Oscar Marchetti, Switzerland; Alexey Maschan, Russia; Malgorzata Mikulska, Italy; Livio Pagano, Italy; Goergios Petrikos, Greece, Daniel Poulain, France; Zdenek Racil, Czech Republic; Pierre Reusser, Switzerland; Patricia Ribaud, France; Malcolm Richardson, UK; Valerie Rizzi-Puechal (Pfizer), France; Markus Ruhnke, Germany; Maurizio Sanguinetti, Italy; Janos Sinko, Hungary; Anna Skiada, Greece; Jan Styczynski, Poland; Anne Thiebaut, France; Paul Verweij, The Netherlands; Claudio Viscoli, Italy; Janice Wahl (Schering-Plough), USA; Katherine Ward, UK; and Philipe White, UK.
